# Assessment of Residual Skeletal and Dental Growth in Children: A Cross-Sectional Study Using Quantitative Cameriere Methods

**DOI:** 10.3390/children13101296

**Published:** 2026-09-24

**Authors:** Raffaella Grimaldi, Barbara Frenna, Giulia Perrotta, Salvatore Fiandaca, Carlo Satta, Stefano Cubeddu, Leonardo Catalini, Roberto Cameriere, Alessio Verdecchia, Enrico Spinas

**Affiliations:** 1 Department of Surgical Sciences, Orthodontics School, University of Cagliari, 09124 Cagliari, Italy; grimaldi.raff@gmail.com (R.G.); barbarafre@outlook.it (B.F.); drgiuliaperrotta@gmail.com (G.P.); fiandaca92@gmail.com (S.F.); drcarlosatta@gmail.com (C.S.); stefanocubeddu98@gmail.com (S.C.); 2 AgEstimation Project, Via E. Bignamini, 60126 Ancona, Italy; leonardo.catalini1994@gmail.com (L.C.); roberto.cameriere@unimol.it (R.C.); 3 Orthodontics Division, Instituto Asturiano de Odontología, Universidad de Oviedo, 33006 Oviedo, Spain

**Keywords:** skeletal maturation, dental maturation, Cameriere method, cervical vertebrae, open apices, orthodontics

## Abstract

**Highlights:**

**What are the main findings?**

• Skeletal and dental maturation showed limited agreement.
• Nearly half of the children showed clinically relevant discrepancies.

**What are the implications of the main findings?**

• A single maturation indicator may not adequately reflect residual growth.
• Combined assessment may improve individualized orthodontic treatment timing.

**Abstract:**

**Background/Objectives:** Accurate assessment of biological maturation is essential in pediatric dentistry and orthodontics for optimizing treatment timing. Quantitative methods proposed by Cameriere allow continuous estimation of residual skeletal and dental growth; however, the degree of agreement between these maturation processes remains unclear. This study aimed to assess residual skeletal and dental growth in children using quantitative Cameriere methods and to evaluate the agreement between these two maturation indicators. **Methods:** This cross-sectional observational study included 98 Caucasian subjects (50 males and 48 females) aged 7.0–11.9 years. Residual skeletal growth was assessed on lateral cephalometric radiographs using the fourth cervical vertebra ratio (C4-Vba), while residual dental growth was evaluated on panoramic radiographs using the open apices method. Measurements were performed using the AgEstimation Project software v1.0. Intra- and inter-observer reproducibility were assessed using intraclass correlation coefficients (ICCs). Correlation analysis and Bland–Altman analysis were performed to evaluate the association and agreement between skeletal and dental maturation. **Results:** Excellent reproducibility was observed for all measurements, with ICC values ranging from 0.93 to 1.00. Residual skeletal growth time (T_skeletal) and residual dental growth time (T_dental) showed a weak positive association (Spearman ρ = 0.18, *p* = 0.09). Discrepancies exceeding the predefined 1-year threshold between skeletal and dental maturation (|ΔT| > 1 year) were observed in 48.0% of subjects. Skeletal predominance was identified in 46.9% of cases, whereas dental predominance was observed in only 1.0%. Females showed significantly greater residual skeletal and dental growth compared with males (*p* < 0.001). **Conclusions:** Skeletal and dental maturation showed limited agreement in children aged 7–11 years, supporting the concept that these processes follow partially independent biological pathways. The integration of quantitative skeletal and dental assessments may improve the evaluation of individual growth potential and support more personalized orthodontic treatment planning.

## 1. Introduction

Accurate assessment of growth and biological maturation is a cornerstone of pediatric dental and orthodontic practice. The effectiveness of many interceptive and orthopedic treatments such as rapid maxillary expansion, functional appliances, extraoral traction, and eruption guidance depends critically on their application during specific biologically favorable time windows [1,2]. Consequently, identifying the residual growth potential of individual patients is essential for optimizing treatment outcomes and minimizing unnecessary or ineffective interventions [3,4]. However, chronological age has consistently been shown to be an unreliable indicator of maturation status. Substantial interindividual variability exists in the timing, velocity, and duration of growth processes, particularly during the prepubertal and pubertal phases [3,5]. This variability limits the clinical utility of age-based approaches and highlights the need for objective biological indicators capable of capturing individual maturation dynamics. Residual skeletal growth assessment has traditionally relied on qualitative methods, such as the cervical vertebral maturation (CVM) method [6,7].

Although widely adopted, these approaches present several limitations, including subjective interpretation, reduced inter-observer reproducibility, and the inability to provide a quantitative estimate of residual growth [7,8]. Several quantitative approaches for skeletal age estimation have been proposed in recent years, including methods based on hand–wrist maturation and cervical vertebral analysis, showing promising applicability in growing subjects [9]. To address these limitations, Cameriere et al. introduced a quantitative approach based on the ratio between the anterior and posterior height of the fourth cervical vertebra (C4-Vba) [10]. This method allows continuous measurement and, through segmented regression models, enables estimation of the residual time to the completion of the pubertal growth spurt. In parallel, dental development has been extensively investigated as a biological indicator of maturation.

Among quantitative dental approaches, the third molar maturity index (I3M) has demonstrated high diagnostic accuracy and reproducibility for biological age assessment and has been extensively validated across different populations and ethnic groups, including the Sardinian population [11,12,13]. These findings further support the value of quantitative radiographic dental indicators for the evaluation of biological maturation. Among the available methods, the approach based on the measurement of open apices in developing teeth has demonstrated high accuracy and reproducibility across different populations [14,15,16]. This method provides a quantitative estimate of dental development and has been widely validated in forensic and clinical contexts across different populations and ethnic groups, including genetically characterized populations such as the Sardinian population [16,17,18,19]. Previous studies have highlighted the relevance of dental age estimation in orthodontic diagnosis and treatment planning, particularly in growing subjects and genetically isolated populations such as Sardinians [16]. Previous studies have reported variable associations between dental and skeletal growth, with substantial heterogeneity across methods and populations [20]. From a biological perspective, skeletal growth is a multifactorial process influenced by genetic and environmental factors [21]. In contrast, dental development appears to have a substantial genetic component [22,23]. These differences in biological regulation may contribute to the limited concordance observed between skeletal and dental maturation indicators in previous studies.

Despite the availability of validated quantitative methods for the independent assessment of skeletal and dental maturation, a significant gap remains in clinical practice: the lack of an integrated approach that allows simultaneous and continuous evaluation of residual growth potential in both compartments. Such an approach could provide clinicians with a more comprehensive understanding of individual maturation patterns and improve decision-making in pediatric and orthodontic treatment planning. Therefore, the aim of this cross-sectional study was to assess residual skeletal and dental growth in children using an integrated quantitative approach and to evaluate the degree of agreement and discrepancy between these two maturation processes.

The primary objective of the present study was to evaluate whether quantitative dental maturation parameters obtained from panoramic radiographs can provide an estimate of residual growth time comparable to that obtained using the quantitative cervical vertebral method. For this purpose, the relationship and agreement between T_dental and T_skeletal were assessed. The clinical rationale was to investigate whether information derived from panoramic radiographs, which are commonly available in orthodontic practice, may provide useful information on residual growth time without requiring an additional radiographic examination specifically for skeletal maturation assessment when this is not otherwise clinically indicated.

## 2. Materials and Methods

### 2.1. Study Design and Setting

This monocentric cross-sectional observational analytical study was conducted at the Orthodontic Department of the AOU of Cagliari, Italy. The retrospective sample selection was conducted between 1 July 2026 and 2 July 2026. Radiographic records were retrospectively collected from patients referred for orthodontic evaluation, using lateral cephalometric and panoramic radiographs previously acquired for routine diagnostic purposes. The study was reported in accordance with the STROBE guidelines [24].

### 2.2. Participants

A total of 186 patients referred for orthodontic evaluation during the study period were initially assessed for eligibility. Following application of the predefined eligibility criteria, 88 patients were excluded, while 98 Caucasian subjects (50 males and 48 females), aged 7.0–11.9 years, were included in the final analysis. The inclusion and exclusion criteria were as follows:

Inclusion criteria:

• Age between 7.0 and 11.9 years;
• Availability of both a pre-treatment panoramic radiograph and a lateral cephalometric radiograph;
• Radiographs of adequate diagnostic quality to allow reliable dental and skeletal maturation measurements;
• Caucasian ethnicity.

Exclusion criteria:

• Previous orthodontic treatment;
• Systemic or syndromic condition;
• Dental anomalies preventing reliable measurement;
• Inadequate radiographic records.

Thus, 88 patients were excluded from the initial sample of 186 patients, and 98 subjects were included in the final analysis. The participant selection process is illustrated in Figure 1.

### 2.3. Sample Size

No a priori sample size calculation or statistical power analysis was performed. Given the retrospective design of the study, the sample size was determined by the number of eligible patients available in the study archive during the predefined study period. All available records meeting the predefined inclusion and exclusion criteria were included. Following the eligibility assessment, 98 subjects were included in the final analysis.

### 2.4. Ethical Approval

This study was approved by the Ethics Committee of Sardinia (CET Sardegna), protocol code 4, session no. 04/2026 of 23 June 2026, and conducted in accordance with the Declaration of Helsinki [25]. All patients participated in the trial on a voluntary basis after receiving comprehensive information about the aim and design of the study and signing an informed consent form.

### 2.5. Radiographic Analysis

All radiographs were obtained for routine diagnostic purposes using a standardized digital radiographic system (Vatech Green X, Vatech Co., Ltd., Hwaseong, Republic of Korea). Lateral cephalometric and panoramic radiographs were acquired according to standardized patient positioning protocols. Measurements were performed using the AgEstimation Project software “https://ageestimation.netlify.app/” (accessed on 6 June 2024), allowing linear measurements, automated parameter calculation, and data export for statistical analysis.

### 2.6. Skeletal Growth Assessment

Skeletal growth was assessed using the quantitative method proposed by Cameriere et al. [10], based on the ratio between the anterior and posterior height of the fourth cervical vertebra (C4), defined as Vba = A/B. All measurements were performed on lateral cephalometric radiographs using the AgEstimation Project software. C4 was identified by counting downward from C2. The anterior (A) and posterior (B) vertebral body heights were measured using digital linear measurement tools. The anterior height was defined as the distance between the superior and inferior anterior points of the vertebral body, while the posterior height was defined as the distance between the superior and inferior posterior points. The ratio between the anterior and posterior heights (Vba = A/B) was calculated for each subject. Sex-specific thresholds derived from segmented regression models were applied (males: Vba_1_ = 0.676, Vba_2_ = 0.966; females: Vba_1_ = 0.658, Vba_2_ = 1.073). For each subject, the residual skeletal growth time (T_skeletal) was estimated as the time remaining until completion of the pubertal growth spurt (corresponding to Vba_2_). Representative vertebral measurements are shown in Figure 2.

### 2.7. Dental Maturation Assessment

Dental maturation was evaluated using the Cameriere method [15] based on the measurement of open apices in the seven left mandibular permanent teeth (Federation Dentaire Internationale 31–37), excluding third molars. All measurements were performed on digital panoramic radiographs using the AgEstimation Project software, a digital platform designed for age estimation analysis. For each tooth with incomplete root development, the apical opening (Ai) was measured as the distance between the inner margins of the open apex, while tooth length (Li) was defined as the distance from the highest point of the crown (incisal edge or cusp tip) to the root apex. In multirooted teeth, Ai was calculated as the sum of the apical openings of individual roots, while in teeth with closed apices Ai was set to zero. Each measurement was normalized (xi = Ai/Li). From these values, the following parameters were derived: the sum of normalized apical openings (s), the number of teeth with closed apices (N_0_), and sex (g, coded as 0 for females and 1 for males). In the present study, the dental parameters were used to estimate the same temporal outcome defined by the cervical vertebral model, namely the residual time to completion of the pubertal growth spurt. Accordingly, T_dental was intended as a dental-based estimate of the residual time to the end of the pubertal growth spurt, rather than as an estimate of chronological age or of the time to completion of dental root development. Residual dental growth time (T_dental) was calculated using the validated regression model: T_dental = 3.88 + 1.10 s − 0.30 N_0_ + 0.57 g. Representative dental measurements are shown in Figure 3A,B.

### 2.8. Outcome Definition

The primary outcome was the difference between skeletal and dental residual growth time, calculated as ΔT = T_skeletal − T_dental. A difference greater than 1 year in absolute value was selected a priori as an operational threshold to identify potentially relevant discrepancies between the two estimates. This threshold was not intended to represent a universally validated clinical cutoff or a specific treatment decision threshold. Accordingly, the resulting categories were considered exploratory and were used to describe the distribution of discrepancies within the study sample. Subjects were classified into skeletal predominance, dental predominance, or concordance according to the direction and magnitude of ΔT.

### 2.9. Reproducibility Analysis

A random subsample of 30 subjects was selected to assess measurement reproducibility. Intra-observer reproducibility was evaluated by repeating measurements after a 4-week interval, while inter-observer reproducibility was assessed by an independent blinded examiner. Agreement was quantified using the intraclass correlation coefficient (ICC) based on a two-way random-effects model for absolute agreement, with 95% confidence intervals [26].

### 2.10. Statistical Analysis

Statistical analysis was performed using R software (version 4.3.2). Data distribution was assessed using the Shapiro–Wilk test and graphical inspection. Normally distributed variables were expressed as mean ± standard deviation (SD), whereas non-normally distributed variables were reported as median and interquartile range (IQR). Categorical variables were presented as frequencies and percentages. Group comparisons were performed using independent-samples *t*-tests or Mann–Whitney U tests, as appropriate. Associations between variables were evaluated using Pearson or Spearman correlation coefficients. Agreement between skeletal and dental maturation was further explored using Bland–Altman analysis. Statistical significance was set at *p* < 0.05. No missing data were observed. To assess proportional bias, linear regression was performed with the difference between residual skeletal and dental growth time as the dependent variable and the corresponding mean of the two measurements as the independent variable. Differences were calculated as ΔT = T_“skeletal” − T_“dental”, whereas means were calculated as M = (T_“skeletal” + T_“dental”)/2. The null hypothesis that the regression slope was equal to zero was tested. The slope, 95% confidence interval, and *p*-value were reported.

## 3. Results

### 3.1. Sample Characteristics

A total of 98 Caucasian subjects (50 males, 48 females) aged between 7.0 and 11.9 years were included in the study. The distribution of the sample across age groups was balanced between sexes (Table 1).

### 3.2. Reproducibility Analysis

All measurements were independently performed by three examiners trained in the application of Cameriere methods. Prior to data collection, a calibration phase was conducted using a subset of radiographs not included in the study sample. Intra-observer reproducibility was assessed by repeating measurements after a 4-week interval for each examiner, while inter-observer agreement was evaluated among the three examiners using the intraclass correlation coefficient (ICC). Reproducibility analysis demonstrated excellent intra- and inter-observer agreement for all parameters. For the C4-Vba ratio, intra- and inter-observer ICC values were 0.97 (95% CI: 0.95–0.98) and 0.93 (95% CI: 0.89–0.96), respectively. For the sum of normalized apical openings (s), the ICC values were 0.985 (95% CI: 0.973–0.991) and 0.962 (95% CI: 0.935–0.979), respectively. Complete agreement was observed for N_0_ (ICC = 1.00) (Table 2).

### 3.3. Residual Skeletal and Dental Growth

Residual skeletal growth time (T_skeletal) showed a non-normal distribution, with a median of 1.81 years (IQR: 1.34–2.45) and a range between 0.87 and 3.73 years. Residual dental growth time (T_dental) also showed a non-normal distribution, with a median of 1.59 years (IQR: 1.09–2.08) and a range between 0.00 and 2.72 years.

### 3.4. Sex Differences

No statistically significant differences were observed between males and females for chronological age, estimated dental age, or Vba values. In contrast, both residual growth indicators were significantly higher in females compared to males (*p* < 0.001) (Table 3). Residual dental growth time differed significantly between males and females, with females showing higher values compared with males (*p* < 0.001) (Figure 4).

Residual skeletal growth time was also significantly greater in females compared with males (*p* < 0.001) (Figure 5).

### 3.5. Correlation Analysis

A weak positive association was observed between T_skeletal and T_dental (Spearman ρ = 0.18, *p* = 0.09).

### 3.6. Discrepancy Exceeding the Predefined 1-Year Threshold

The difference between skeletal and dental maturation (ΔT) showed an approximately normal distribution (Shapiro–Wilk *p* = 0.08) with a mean of 0.86 ± 0.99 years. A discrepancy exceeding the predefined 1-year threshold. (|ΔT| > 1 year) was observed in 47 subjects (48.0%), whereas 51 subjects (52.0%) showed concordance between skeletal and dental maturation. Among discordant cases, 46 subjects (46.9%) showed skeletal predominance, while only one subject (1.0%) showed dental predominance (Table 4). Bland–Altman analysis showed a mean difference (bias) of 0.86 years, with wide limits of agreement, indicating substantial variability between skeletal and dental maturation estimates.

Agreement between residual skeletal and dental growth estimates was further evaluated using Bland–Altman analysis (Figure 6). The mean difference (bias) between the two methods was 0.86 years, with 95% limits of agreement ranging from −1.06 to 2.77 years. Regression of the differences between T_skeletal and T_dental on their corresponding means showed a statistically significant negative slope (β = −0.55, 95% CI: −0.891 to −0.209; *p* = 0.002). This finding indicated evidence of proportional bias, with the difference between the two estimates tending to decrease as the mean residual growth time increased.

## 4. Discussion

The present cross-sectional study evaluated residual skeletal and dental growth in a pediatric population using two validated quantitative methods and explored their degree of agreement. The main findings were threefold: (i) both methods demonstrated excellent reproducibility, (ii) skeletal and dental maturation showed a weak and non-significant association, and (iii) a substantial proportion of subjects exhibited discrepancies exceeding the predefined 1-year threshold between the two indicators. The reproducibility analysis confirmed the robustness of both the C4–Vba ratio and Cameriere’s dental method. The high intra- and inter-observer agreement observed in this study is consistent with previous reports and supports the use of quantitative approaches as reliable alternatives to traditional qualitative methods [8,10,26]. The use of standardized digital tools likely contributed to minimizing measurement error and improving consistency. Regarding growth assessment, both skeletal and dental residual growth times showed wide variability, reflecting the biological heterogeneity typical of the prepubertal phase. The age range of 7.0–11.9 years was selected to include children undergoing active skeletal and dental maturation, allowing residual skeletal and dental growth time to be quantitatively estimated and compared within the same individuals. We acknowledge that younger children, particularly those aged 7–9 years, generally have a greater amount of residual skeletal growth and may therefore present greater variability in the estimated remaining growth time. However, the aim of the present study was not to predict the exact timing of the pubertal growth spurt, but to investigate the relationship and degree of discrepancy between quantitative skeletal and dental estimates of residual growth. Therefore, the inclusion of this age range was considered appropriate for addressing the specific research question. Nevertheless, the greater residual growth expected in younger subjects should be considered when interpreting the findings. Notably, residual skeletal growth tended to exceed dental growth in most subjects, as evidenced by the predominance of positive ΔT values. This finding suggests that skeletal growth may progress at a slower rate compared with dental development within this age range. Sex-related differences were also observed. Although chronological age, dental age, and Vba values were comparable between males and females, both T_skeletal and T_dental were significantly higher in females. These findings may reflect differences in the timing and dynamics of skeletal and dental maturation between sexes. However, the present cross-sectional design does not allow the underlying biological mechanisms of these differences to be determined, nor does it permit direct inference regarding individual growth trajectories. Therefore, this interpretation should be considered hypothesis-generating and requires confirmation in longitudinal studies [5]. A key finding of this study is the weak and non-significant correlation between skeletal and dental residual growth. The previous literature has reported an overall positive but heterogeneous relationship between dental and cervical vertebral maturation, with very low certainty of evidence [20]. Therefore, the present findings support interpreting the two indicators as complementary rather than interchangeable. The present study was not designed to replace the Cervical Vertebrae Maturation method with the Cameriere approach. Rather, it aimed to determine whether quantitative dental parameters could provide a dental-based estimate of the same temporal outcome, namely residual growth time. Therefore, the Cameriere method should be interpreted as a complementary quantitative approach rather than as a substitute for skeletal maturation assessment. From a biological perspective, skeletal growth and dental development are influenced by overlapping but distinct biological determinants. Skeletal growth is affected by genetic, endocrine, nutritional, and environmental factors [21], whereas dental development is also substantially influenced by genetic determinants, although environmental and systemic factors may contribute as well [22,23]. These differences in the relative contribution of biological factors may contribute to the limited concordance observed between the two indicators; however, the present study does not allow the specific mechanisms underlying this association to be determined. Importantly, nearly half of the sample (48.0%) showed a discrepancy exceeding the predefined 1-year threshold between skeletal and dental residual growth estimates. Among these cases, skeletal predominance was overwhelmingly more frequent than dental predominance. This asymmetry may suggest that dental and skeletal indicators can provide different information regarding residual growth timing. However, whether reliance on dental indicators alone results in clinically meaningful underestimation of residual skeletal growth potential cannot be established from the present cross-sectional data. From a clinical perspective, these findings may have potential implications for orthodontic growth assessment, as skeletal and dental maturation may provide complementary information regarding individual biological timing. However, the present study did not evaluate treatment outcomes or compare treatment strategies based on single versus combined maturation indicators. Therefore, whether integrating skeletal and dental maturation assessments improves treatment timing or clinical outcomes remains to be established in prospective longitudinal studies. In this context, quantitative methods allowing continuous estimation of residual growth represent a significant advantage over traditional staging systems [6,10]. Several limitations should be acknowledged. The cross-sectional design does not allow direct observation of growth changes over time and limits causal inference. Moreover, the quantitative estimate of residual skeletal growth time should not be interpreted as a direct measurement or exact prediction of future individual growth. The C4-Vba method derives the estimated residual growth time from radiographic measurements and regression models and may therefore be influenced by biological variability in the timing and magnitude of skeletal growth. Because of the cross-sectional design of the present study, the estimated residual growth time could not be prospectively compared with the subsequent growth actually observed in each participant. Therefore, the estimates obtained should be interpreted as quantitative indicators of biological timing rather than as exact predictions of individual growth trajectories. Longitudinal studies are needed to evaluate the agreement between estimated residual growth and subsequent observed growth. The sample consisted of children referred for orthodontic evaluation at a single institution and therefore represents a clinical rather than population-based sample. Consequently, the possibility of selection bias cannot be excluded, as children undergoing orthodontic assessment may differ from healthy children in the general population in terms of dentofacial characteristics or indications for referral. Therefore, the present sample should not be considered fully representative of the general healthy pediatric population aged 7.0–11.9 years, and the generalizability of the findings should be interpreted accordingly. A further limitation is that no a priori sample size calculation or statistical power analysis was performed, as the final sample size was determined by the availability of eligible records in the study archive. Therefore, the possibility of limited statistical power for detecting smaller associations cannot be excluded. The sample was also restricted to Caucasian subjects, which may further limit generalizability to other populations [16,17,18,19,23]. Furthermore, although validated quantitative methods were used, measurements were based on radiographic images and may be influenced by image quality and anatomical variability.

## 5. Conclusions

This study demonstrates that skeletal and dental maturation, when assessed using Cameriere’s quantitative methods, show limited agreement in children aged 7 to 11 years. A weak association between skeletal and dental residual growth was observed, and nearly half of the subjects exhibited discrepancies exceeding the predefined 1-year threshold, predominantly characterized by greater residual skeletal growth. These findings support the concept that skeletal and dental development may follow partially independent biological pathways and should not be considered interchangeable in clinical practice. Reliance on a single indicator may provide an incomplete representation of individual biological maturation. The integration of quantitative skeletal and dental assessments may provide complementary information for the evaluation of individual maturation status and may have potential implications for personalized treatment planning. However, a clinical benefit of integrating these assessments was not demonstrated in the present study. Future research should focus on longitudinal studies to validate the predictive value of combined maturation indicators and to explore their role in clinical decision-making. The development of integrated predictive models incorporating multiple biological markers may further enhance the assessment of individual growth potential.

## Figures and Tables

**Figure 1:**
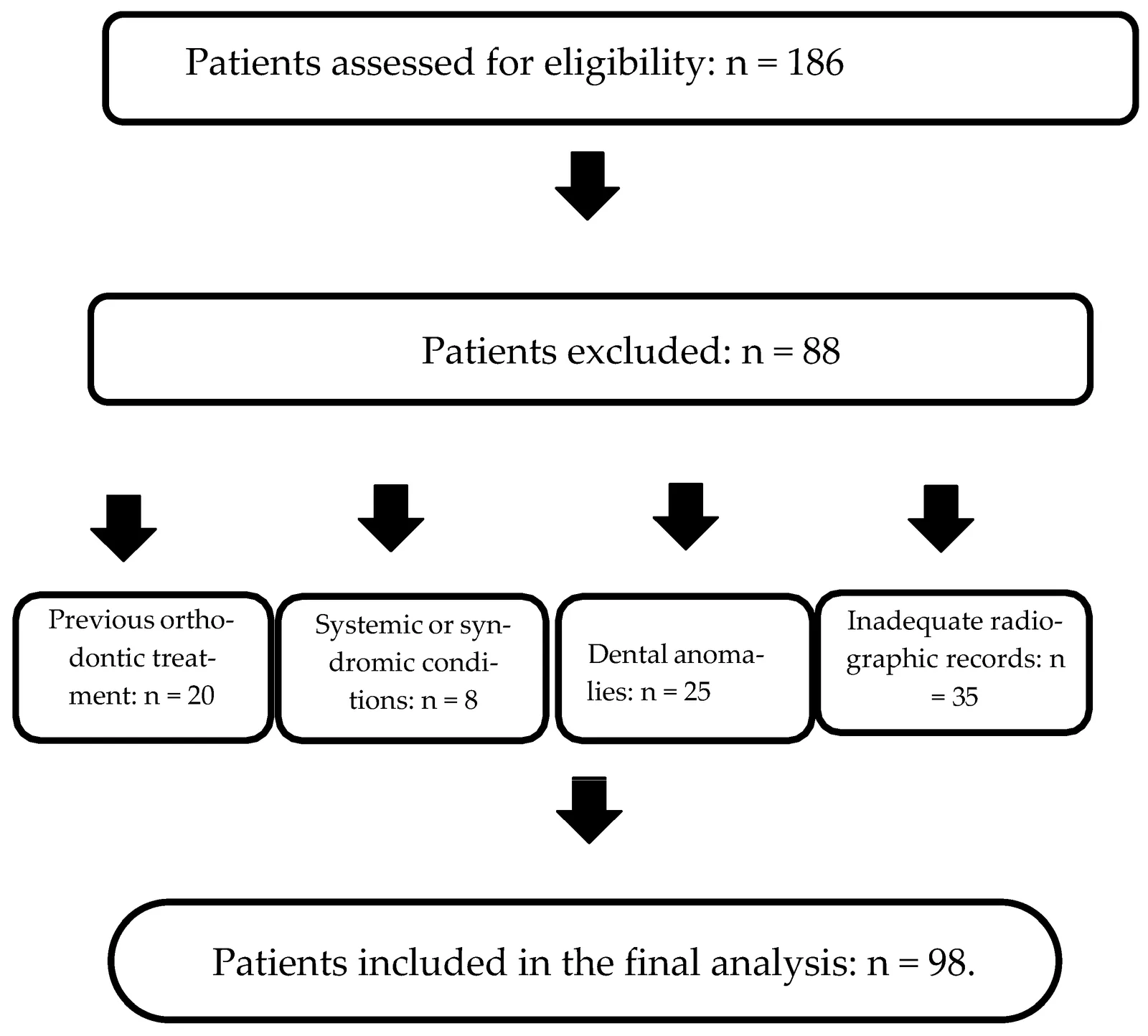
Flow diagram of participant selection and inclusion in the study.

**Figure 2:**
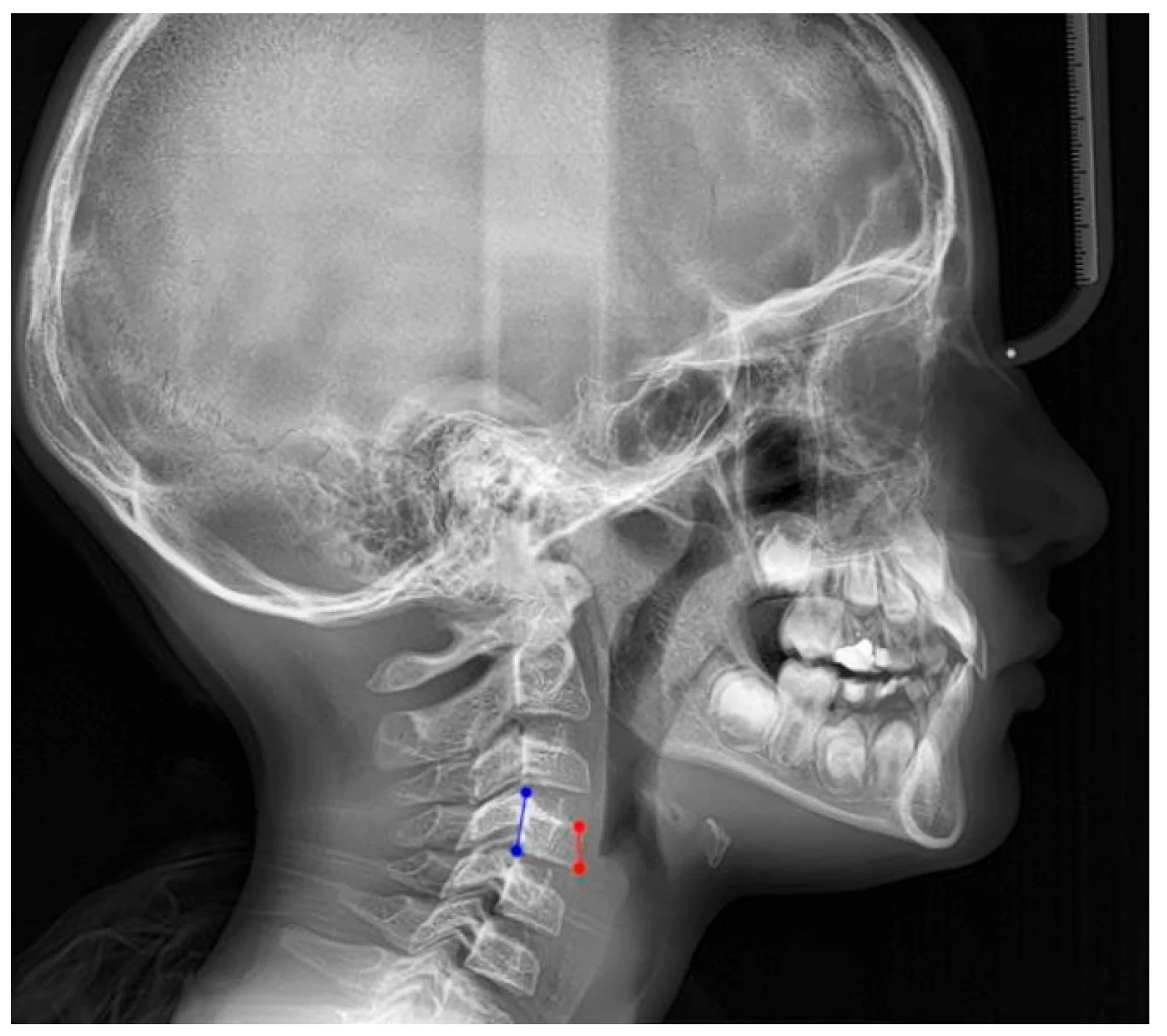
Measurement of the anterior (red line) and posterior (blue line) heights of the fourth cervical vertebra (C4) on lateral cephalometric radiograph for calculation of the Vba ratio.

**Figure 3:**
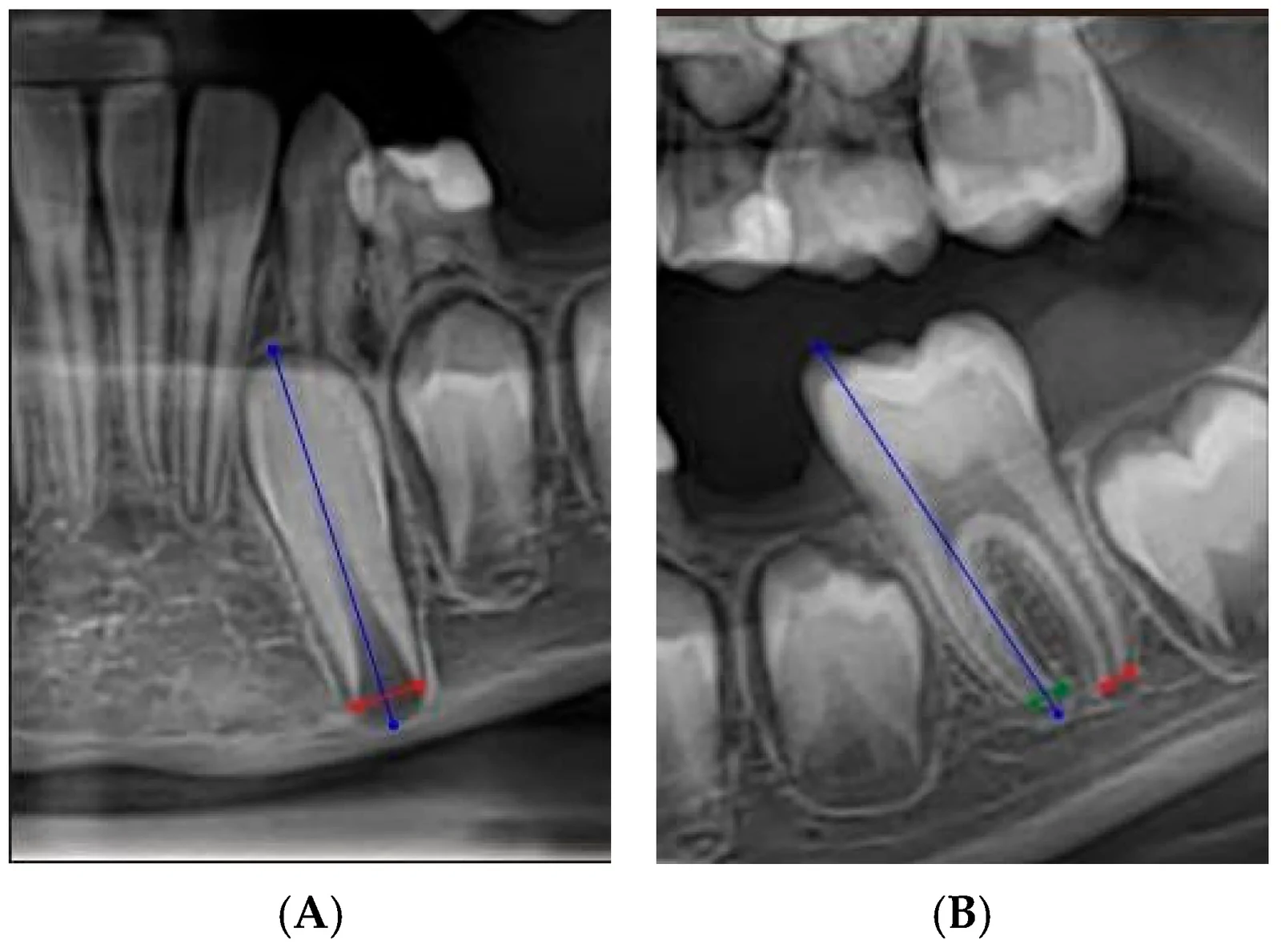
Measurement of tooth length (blue line) and apical opening (red line) according to the Cameriere method: (**A**) measurements performed on the single-rooted tooth 33; (**B**) measurements performed on the multirooted tooth 36.

**Figure 4:**
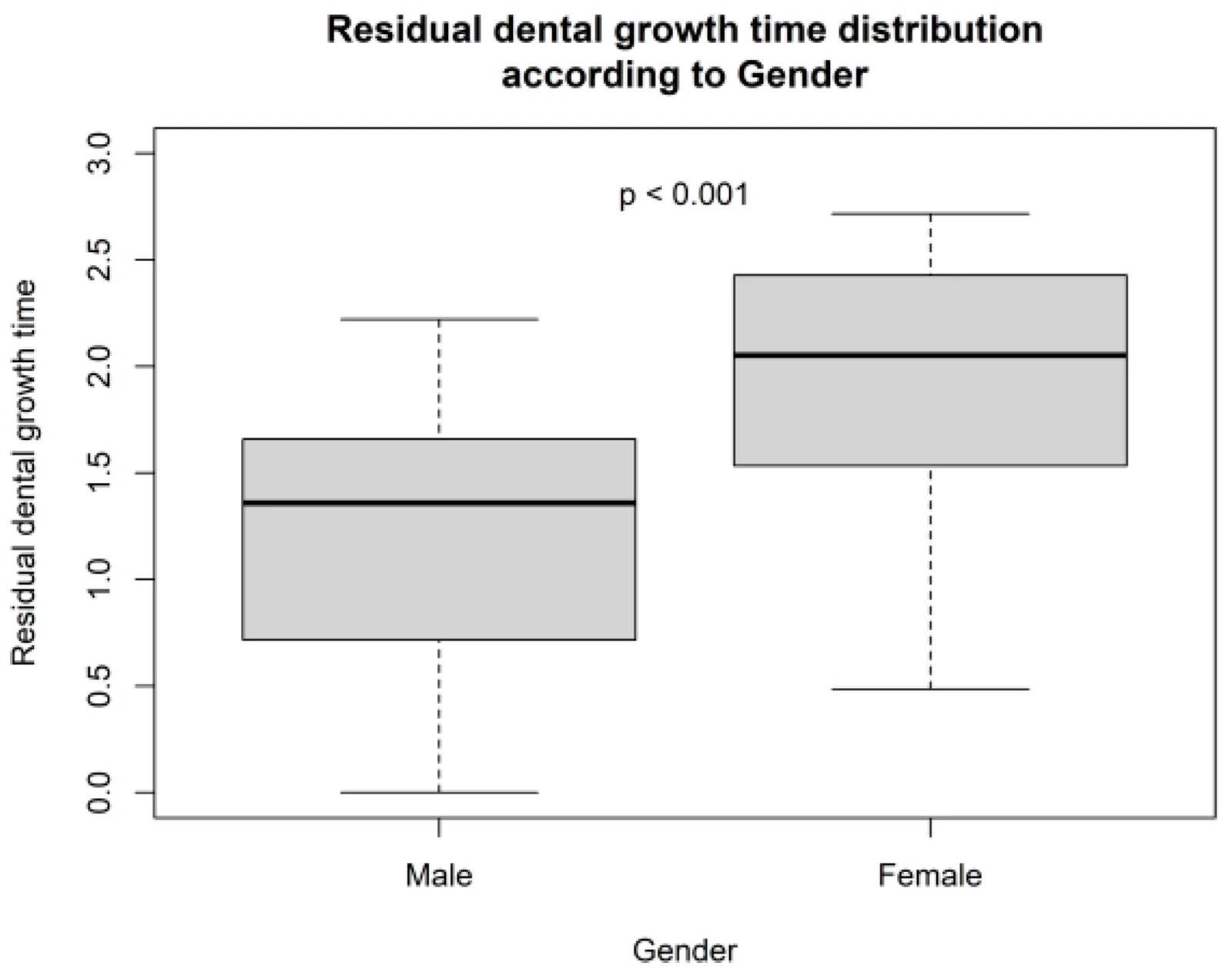
Boxplot showing the distribution of residual dental growth time according to gender.

**Figure 5:**
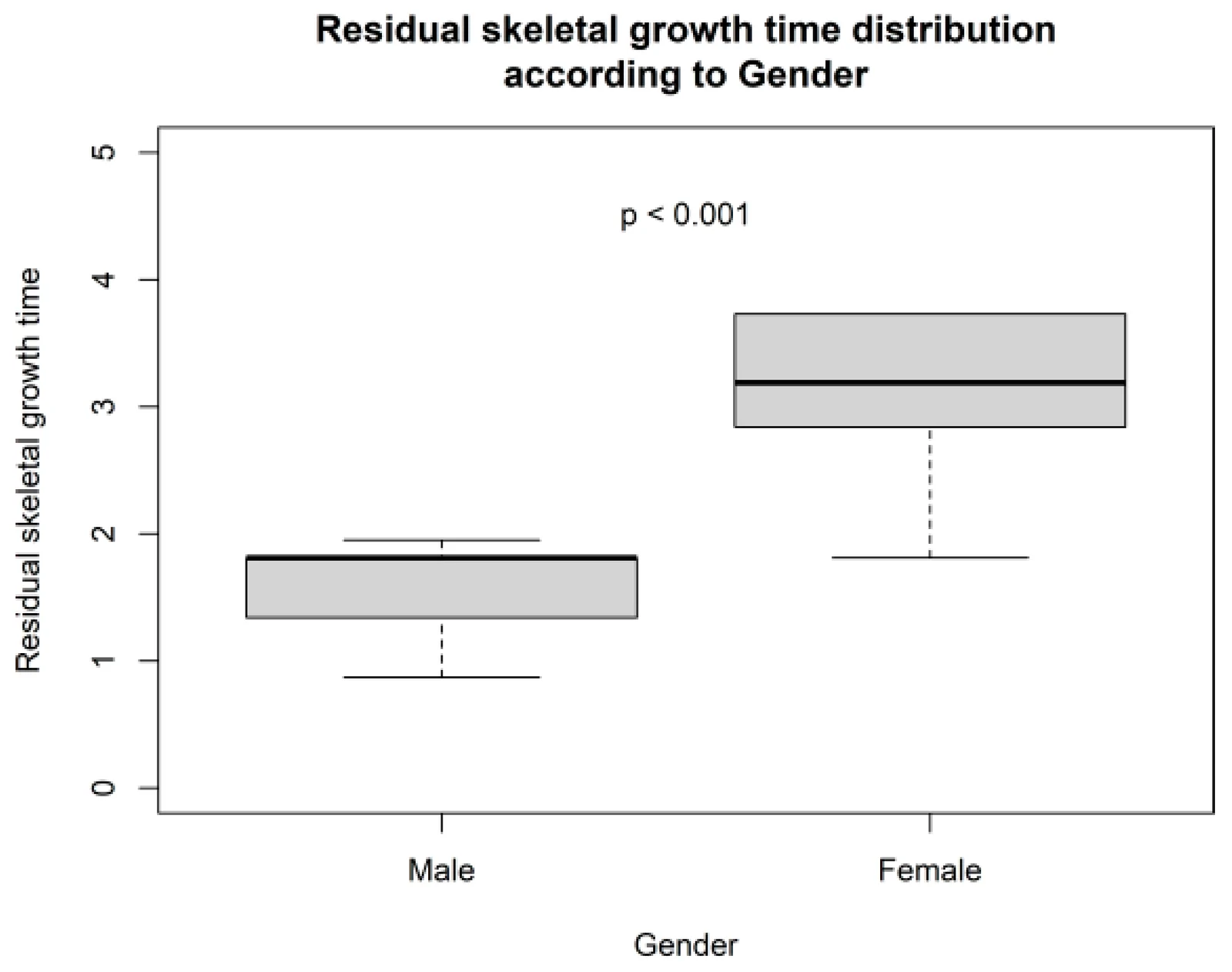
Boxplot showing the distribution of residual skeletal growth time according to gender.

**Figure 6:**
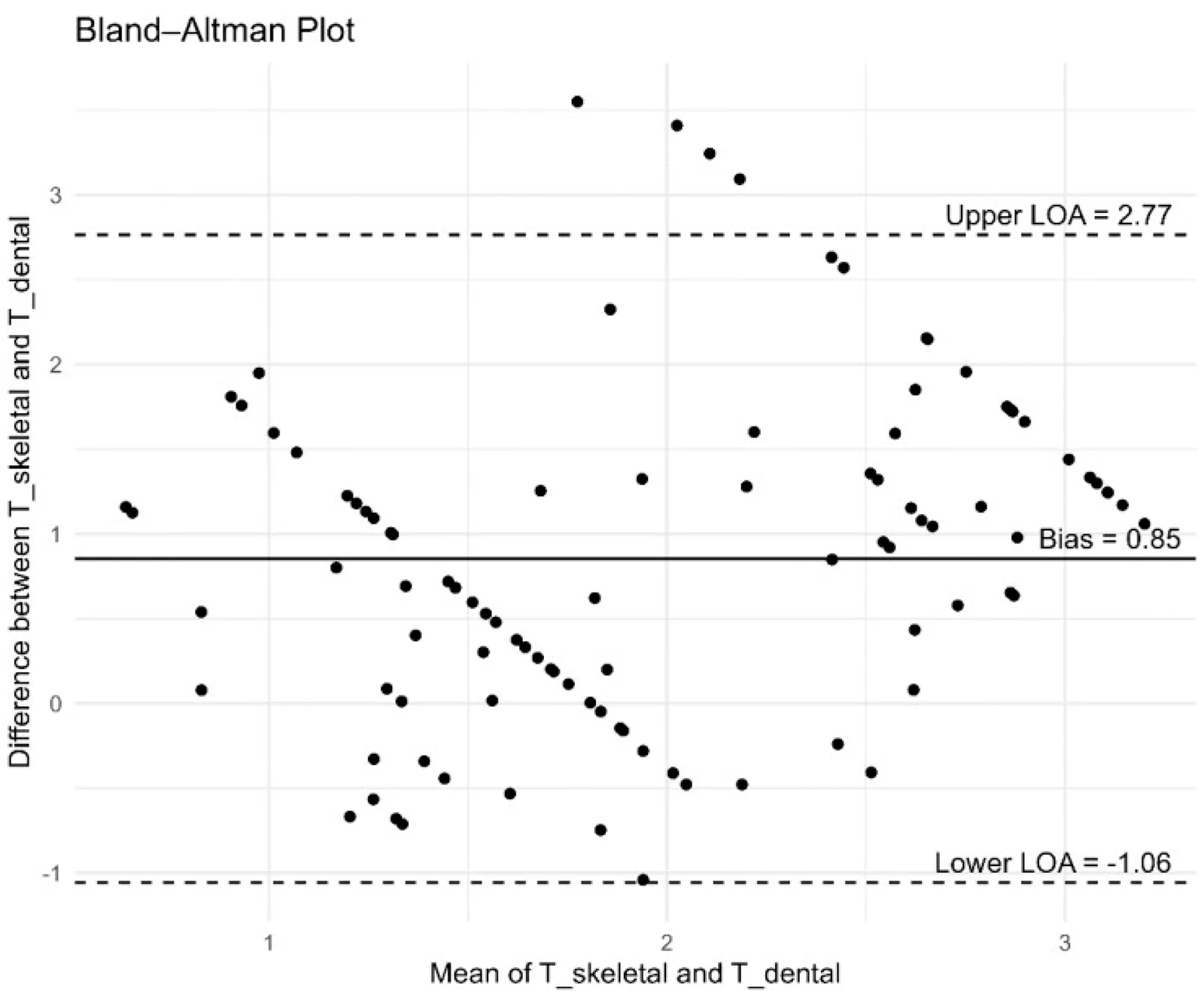
Bland–Altman plot showing agreement between residual skeletal growth time (T_skeletal) and residual dental growth time (T_dental). The solid line represents the mean difference (bias), while dashed lines indicate the 95% limits of agreement.

**Table 1:** Distribution of the study sample by age and sex (n = 98).

Age Group (Years)	Females n %	Males n %	Total n %
7.0–7.9	10 (20.8)	8 (16.0)	18 (18.4)
8.0–8.9	10 (20.8)	12 (24.0)	22 (22.4)
9.0–9.9	10 (20.8)	10 (20.0)	20 (20.4)
10.0–10.9	10 (20.8)	10 (20.0)	20 (20.4)
11.0–11.9	8 (16.7)	10 (20.0)	18 (18.4)
Total	48 (49.0)	50 (51.0)	98 100)

**Table 2:** Intra- and inter-observer reproducibility.

Parameter	ICC Intra (95% CI)	ICC Inter (95% CI)
Vba	0.97 (0.95–0.98)	0.93 (0.89–0.96)
S	0.985 (0.973–0.991)	0.962 (0.935–0.979)
N_0_	1.00 (1.00–1.00)	1.00 (1.00–1.00)

Abbreviations: Vba, ratio between anterior and posterior height of the fourth cervical vertebra; S, sum of normalized apical openings; N_0_, number of teeth with closed apices; ICC, intraclass correlation coefficient; CI, confidence interval.

**Table 3:** Comparison between males and females.

Variable	Males	Females	*p*-Value
Chronological age	9.00 (8.00–10.11)	9.00 (8.00–10.00)	0.760
Dental age	8.73 (7.80–10.15)	8.90 (7.75–10.09)	0.994
Vba	0.67 ± 0.09	0.69 ± 0.10	0.535
T_dental	1.36 (0.73–1.65)	2.05 (1.55–2.43)	<0.001
T_skeletal	1.81 (1.34–1.81)	3.19 (2.84–3.73)	<0.001

Abbreviations: Vba, ratio between anterior and posterior height of the fourth cervical vertebra; T_dental, residual dental growth time; T_skeletal, residual skeletal growth time.

**Table 4:** Discrepancy exceeding the predefined 1-year threshold classification.

Category	n	%
Skeletal predominance	46	46.9
Dental predominance	1	1.0
Concordance	51	52.0
Total	98	100

## Data Availability

The data presented in this study are available on request from the corresponding author. Data are unavailable due to the privacy of the patients.
